# Comparative proteomic analysis implicates eEF2 as a novel target of PI3Kγ in the MDA-MB-231 metastatic breast cancer cell line

**DOI:** 10.1186/1477-5956-11-4

**Published:** 2013-01-15

**Authors:** Meizhi Niu, Manuela Klingler-Hoffmann, Julie A Brazzatti, Briony Forbes, Chareeporn Akekawatchai, Peter Hoffmann, Shaun R McColl

**Affiliations:** 1 School of Molecular and Biomedical Science, University of Adelaide, Adelaide, SA 5005, Australia; 2 Current address: Department of Medical Technology, Thammasat University, Patumtani, 121212, Thailand; 3 Current address: Immunology Group, Paterson Institute for cancer research, The University of Manchester, Manchester, M20 4BX, England

**Keywords:** Receptor transactivation, Cell migration, IGF-I, CXCR4, PI3Kγ, eEF2, 2D-DIGE

## Abstract

**Background:**

Cancer cell migration is fundamentally required for breast tumour invasion and metastasis. The insulin-like growth factor 1 tyrosine kinase receptor (IGF-1R) and the chemokine G-protein coupled receptor, CXCR4 have been shown to play an important role in breast cancer metastasis. Our previous study has shown that IGF-1R can transactivate CXCR4 via a physical association in the human MDA-MB-231 metastatic breast cancer cell line and that this plays a key role in IGF-I-induced migration of these cells. In the present study we used pharmacological inhibition and RNAi to identify PI3Kγ as an important migration signalling molecule downstream of receptor transactivation in MDA-MB-231 cells. To identify PI3Kγ-regulated proteins upon transactivation of CXCR4 by IGF-I, we undertook a comparative proteomics approach using 2-D- Fluorescence Difference Gel Electrophoresis (DIGE) and identified the proteins by mass spectrometry.

**Results:**

These experiments identified eukaryotic elongation factor 2 (eEF2) as a novel downstream target of PI3Kγ after activation of the IGF-1R-CXCR4 heterodimer by IGF-I. Further analysis demonstrated that eEF2 is phosphorylated in MDA-MB-231 cells in response to IGF-I and that this is dependent on PI3Kγ activity.

**Conclusions:**

Our data imply a novel role for PI3Kγ in facilitating cell migration by regulating phosphorylation of eEF2.

## Background

Breast cancer metastasis is a multi-step process regulated by a number of homeostatic factors including chemokines and growth factors through interaction with their corresponding receptors, G protein-coupled receptors (GPCRs) and tyrosine kinase receptors (RTKs) respectively. A number of studies have shown that the signaling pathways initiated by these receptors are not activated in a linear way and instead involve activation of interacting signaling networks. For instance, in bladder cancer cells, it has been shown that LPA promotes cell migration and invasion via phosphorylation of EGFR and subsequent activation of mitogen-activated protein kinase (MAPK) signalling [1]. Recent evidence indicates an additional level of complexity in these systems: receptor heterodimerization whereby transactivation between two distinct receptors occurs [2]. Our previous data have demonstrated that insulin-like growth factor 1 receptor (IGF-1R) can transactivate the chemokine receptor CXCR4 via a physical association between IGF-1R and CXCR4 in human MDA-MB-231 metastatic breast cancer cells and that this plays a key role in IGF-I-induced motility of these cells [2]. Furthermore, RNAi-mediated knockdown of CXCR4 in these cells prevents experimental metastasis [3]. Therefore cancer metastasis appears to depend on CXCR4 and the signalling occurring downstream of this receptor. However, the downstream signaling events occurring as a result of this transactivation are yet to be elucidated.

Phosphoinositide-3-kinases (PI3Ks) have been demonstrated to be critical in cell migration downstream of both GPCRs [4] and RTKs [5,6]. PI3Ks are grouped into three classes according to sequence homology, substrate preference and tissue distribution [7]. The most extensively investigated PI3Ks, class I PI3Ks are further divided into class IA and class IB. Class IA PI3Ks, including PI3Kα, PI3Kβ and PI3Kδ are mainly activated by RTKs while the class IB PI3K, known as PI3Kγ is activated by GPCRs [8] although PI3Kδ has also been shown to be activated downstream of GPCRs notably, CXCR4 [9]. Class I PI3Ks phosphorylate the 3’-OH group on phosphatidylinositols in the plasma membrane, leading to the recruitment and activation of adaptor and effector proteins containing a pleckstrin homology (PH) domain. This triggers a series of downstream signaling cascades to regulate survival, metabolism, growth and migration [10], which can be inhibited by treatment with the well-characterized pan-PI3K inhibitors wortmannin [11] and LY294002 [12]. Furthermore, a number of recent studies have demonstrated distinct roles of specific PI3K isoforms in response to GPCR and RTK activation in a variety of cancer cell lines, including breast cancer cells,using isoform-specific inhibitors. For instance, it has been shown that PI3Kδ is the most important class IA PI3K in the regulation of EGF-driven motility of breast cancer cells, whereas PI3Kβ is required for directed migration but PI3Kα does not appear to play a role in breast cancer cell migration [13]. With respect to the class IB PI3K, it has been demonstrated that PI3Kγ regulates LPA-induced Akt activity and cell proliferation in pancreatic cancer cells [14] and also plays an important role in cell invasion and adhesion of melanoma cells in response to CXCL12 [15]. However, the role of specific PI3K isoforms in receptor transactivation has not been investigated.

To elucidate the underlying mechanism by which IGF-1R-CXCR4 transactivation regulates cell migration, we investigated signaling transduction pathways activated downstream of IGF-I-induced activation of IGF-1R-CXCR4 heterodimers in invasive MDA-MB-231 cells, focusing particularly on the role of PI3Ks and subsequent downstream effectors. The experimental design for this study is outlined in Additional file 1: Figure S1. Our results show that PI3Kγ is the major class I PI3K isoform regulating cell migration in response to ligation of IGF-1R-CXCR4 heterodimers by IGF-I in MDA-MB-231 cells.After we had established the central role of PI3Kγ in regulation of migration downstream of transactivation, we wanted to identify downstream effectors of PI3Kγ. We identified eukaryotic elongation factor 2 (eEF2) as one of the downstream targets that are dependent on PI3Kγ activation using 2D Fluorescence Difference Gel Electrophoresis (DIGE) and mass spectrometry analysis. Phosphorylation of eEF2 on Thr56 decreases it affinity to the ribosome, thereby inhibiting elongation, a key step in the process of translating mRNA. Recently, it has been shown, that phosphorylation of eEF2 facilitates inhibition of protein synthesis downstream of DNA damage [16].Thus, our findings imply that PI3Kγ facilitates breast cancer cell migration through a novel mechanism by deactivating eEF2, thereby inhibiting protein synthesis after IGF-1R-CXCR4 transactivation.

## Results

### Phosphorylation of Akt is dependent on CXCR4

**Figure 1:**
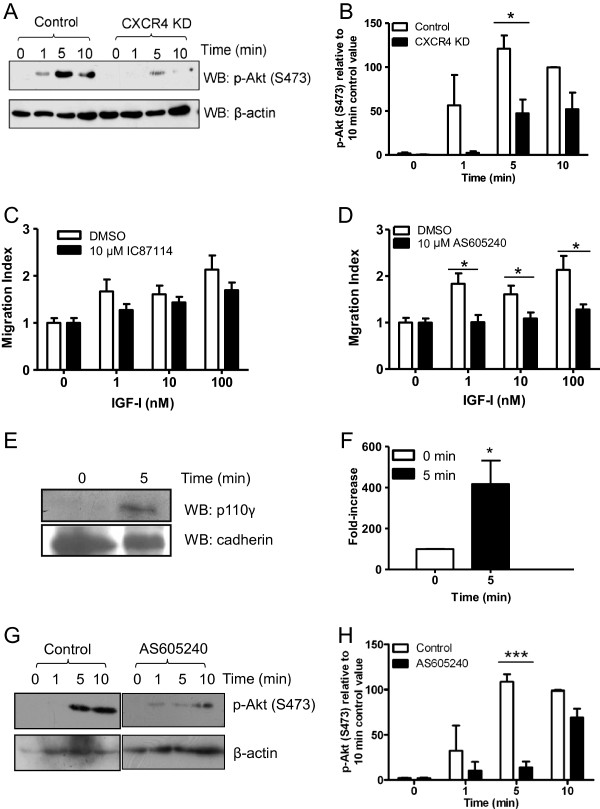
**PI3Kγ plays a key role in IGF-I-induced migration of MDA-MB-231 cells.** (**A**) MDA-MB-231 cells or MDA-MB-231 CXCR4 knock-down cells were treated with 0.1 nM IGF-I and Akt phosphorylation was assessed. (**B**) Akt phosphorylation was quantified by densitometry, normalized to β-actin and expressed as a value relative to the 10-minute control values. (**C**) MDA-MB-231 cells were treated with DMSO (diluent control) or 10 μM IC87114 for 1 hour and chemotaxis in response to IGF-I assessed. (**D**) MDA-MB-231 cells were treated with diluent or 2 μM AS605240 for 1 hour and chemotaxis in response to IGF-I assessed. * - significantly different from the control values at, p<0.05 (**E**) MDA-MB-231 cells were incubated in serum-free medium for 1 hour and stimulated with 0.1 nM IGF-I for 5 min. Cell membrane fractions were analyzed by SDS-PAGE and Western blot using anti-p110γ antibody. The Western-blots were stripped and reprobed with anti-pan-cadherin antibodies as a loading control. (**F**) Membrane translocation of p110γ was quantified by densitometry analysis of three independent experiments. * - significantly different from the control values at, p<0.05 (G) MDA-MB-231 cells were either treated with diluent or 2 μM AS605240 for 1 hour and Akt phosphorylation was assessed. (**H**) Akt phosphorylation was quantified by densitometry, normalized to β-actin and expressed as a value relative to the 10-minute control-treated values. * - significantly different from the control values (2-way ANOVA with Bonferroni post-test) at ***, p<0.001. Unless otherwise stated, data are expressed as mean ± sem from at least three experiments.

Our previous results have shown that migration of MDA-MB-231 is dependent on CXCR4 expression, because knock-down of CXCR4 led to reduced migration upon IGF-I stimulation [17]. Therefore, we determined if activation of PI3K occurs downstream of CXCR4 following stimulation of the cells with IGF-I. To achieve this, we used the phosphorylation of Akt on S473 as a readout for PI3K activity. Knock-down of CXCR4 nearly completely abrogated the phosphorylation of Akt in MDA-MB-231 cells upon stimulation with IGF-I (Figure 1A and B).

### Treatment with the selective PI3Kγ inhibitor suppresses IGF-I-induced migration

The two major PI3K indicated downstream of G-protein coupled receptors are PI3Kγand PI3Kδ. Both are expressed in MDA-MB-231 cells as determined by Western-Blot analysis (data not shown). To determine if PI3Kγ or PI3Kδ play a role in IGF-I-induced migration of MDA-MB-231 cells, the cells were pre-treated with specific inhibitors, as described previously [18] and cell migration in response to IGF-I was determined using a modified Boyden chamber assay. The inhibition efficiency of the selective PI3Kδ inhibitor IC87114 was first examined. MDA-MB-231 cells migrated towards IGF-I in a dose-dependent manner and this was only slightly inhibited when cells were pretreated with 10 μM IC87114 (Figure 1C). In contrast migration was completely abrogated after preincubation with 10 μM AS605240, the selective PI3Kγ inhibitor (Figure 1D). Lower levels of inhibitor treatment (2 μMAS605240) also led to a significant inhibition in migration upon stimulation with 100 nM IGF-I. This involvement of PI3Kγ in IGF-I-induced cell migration was dependent on CXCR4 transactivation as AS605240 had no effect on IGF-I-induced MDA-MB-231 cell migration in CXCR4 knockdown cells (data not shown). Moreover, AS605240 treatment had no effect on IGF-I-induced migration of MCF-7 cells, which do not express a functional IGF-1R-CXCR4 heterodimer [2] (data not shown). We conclude that PI3Kγ most likely regulates migration in MDA-MB-231 cells downstream of IGF-I stimulation and requires CXCR4 transactivation.

### The p110γ catalytic subunit translocates to the membrane after IGF- stimulation

To provide further evidence that IGF-I activates PI3Kγ in MDA-MB-231 cells, the translocation of the p110γ to the membrane was investigated [19]. MDA-MB-231 cells were incubated with IGF-I for 5 minutes and membrane fractions were compared for the presence of p110γ by Western blot analysis (Figure 1E). Three independent experiments were analysed by densitometry (Figure 1F). The results of these experiments clearly indicate that IGF-I induces translocation of p110γ to the membrane.

### Treatment with the selective PI3Kγ inhibitor suppresses phosphorylation of Akt

One of the earliest detectable events downstream of PI3K is phosphorylation of Akt/PKB [20]. In fact, Akt phosphorylation on S473 is commonly used as surrogate readout of PI3K activation. Therefore, Akt phosphorylation upon IGF-1R-CXCR4 transactivation in response to IGF-I was investigated by Western blot analysis using phospho-Akt antibody. Cells, either untreated or treated with 2 μM of AS605240 were stimulated with IGF-I and the lysates were immunoblotted with phospho-Akt antibody. The levels of phospho-Akt were significantly decreased after AS605240 treatment (Figure 1G). Three independent experiments were analysed by densitometry (Figure 1H). In summary, these data indicate that PI3Kγ is the major isoform regulating phosphorylation of Akt and migration downstream of IGFR-CXCR4 transactivation.

### Effect of p110γ knock-down on IGF-I-induced transactivation of CXCR4

**Figure 2:**
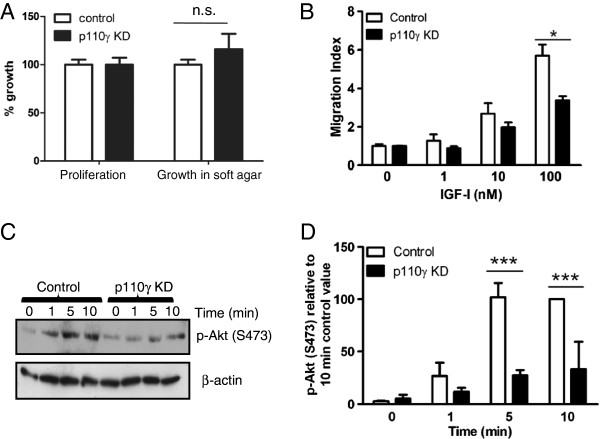
**Knock-down of p110γ inhibits cell migration and phosphorylation of Akt in MDA-MB-231 cells after IGF stimulation.** (**A**) MDA-MB-231 cells in which p110γ had been knocked down by RNAi, or control knockdown cell (expressing scrambled RNAi) were assessed for growth in culture for 5 days, or growth in soft agar for 2 weeks. (**B**) Control and p110γ knockdown cells were tested for their chemotactic response to various concentrations of IGF-I. All panels are expressed as the mean ± SEM from at least three separate experiments, each performed in triplicate. Asterisks indicate significantly different from the control values (Student’s unpaired t test) at *, p<0.05. (**C**) Akt phosphorylation on S473 of MDA-MB-231 cells or p110γ knock-down cells was assessed in response to 0.1 nM IGF-I at the time points indicated. These data are representative of at least 3 independent experiments conducted with similar results. (**D**) Akt phosphorylation was quantified by densitometry, normalized to the level of β-actin and expressed as a value relative to the 10 minute control-treated values (mean ± SEM of three independent experiments) as described in Materials and Methods. Asterisks indicate significantly different from the control values (2-way ANOVA with Bonferroni post-test) at ***, p<0.001.

To further investigate the role of PI3Kγ in IGF-I-induced transactivation of CXCR4, p110γ was knocked down in MDA-MB-231 cells as previously reported [17]. RNAi-mediated knockdown of p110γwas achieved to approximately 85% and had no effect in cell proliferation or growth in soft-agar assays (Figure 2A). Thus, the effect of knockdown of p110γ on IGF-I-induced MDA-MB-231 cell migration and Akt phosphorylation was examined. Both migration of MDA-MB-231 cells (Figure 2B) and phosphorylation of Akt (Figures 2C & D) in response to IGF-I were significantly inhibited by knockdown of p110γ (Figure 2B). Taken together, these results confirm that PI3Kγ is required for IGF-I-induced migration of MDA-MB-231 cells, and this is dependent on transactivation of CXCR4 by IGF-1R.

### Proteomic analysis

**Figure 3:**
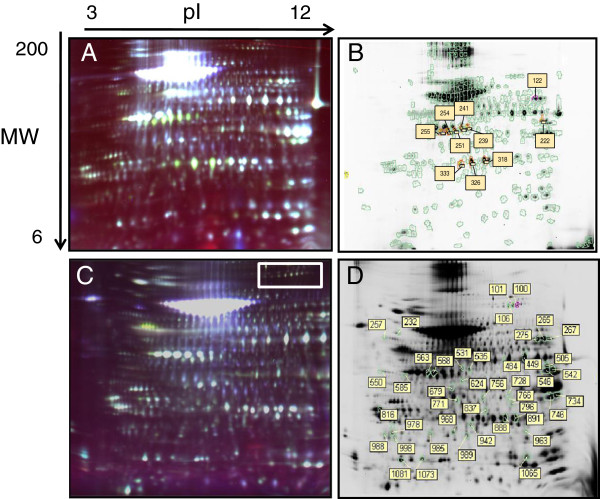
**Representative overlay 2D gels images of MDA-MB-231 cells and p110γ KD cells.** (**A**) Overlay gel image of the cytosolic proteome of MDA-MB-231 cells and p110γ knockdown cells without stimulation. Cytosolic fractions from triplicate samples were labeled with Cy3 or Cy5 while the pool of these six samples was labeled with Cy2 as the internal pooled standard. These labeled samples were combined and subjected to 2D gel separation, followed by scanning at 100 μm resolution using a Typhoon Trio. These data are representative of 3 gels run with similar results. (**B**) Cy5-labeled representative gel of the protein fractions from p110γ knockdown cells. Number-labeled rectangles indicate the protein spots with altered abundance. (**C**) Overlay gel image of the cytosolic proteome of MDA-MB-231 cells and p110γ knockdown cells after 5 min stimulation with IGF-1. Cytosolic fractions from triplicate samples were labeled with Cy3 or Cy5 while the pool of these six samples was labeled with Cy2 as the internal pooled standard. These labeled samples were combined and subjected to 2D gel separation, followed by scanning at 100 μm resolution using a Typhoon Trio. These data are representative of 3 gels run with similar results. The white rectangle indicates the area containing the spots 101, 102 and 106. (**D**) Cy5-labeled representative gel of the protein fractions from p110γ knockdown cells. Number-labeled rectangles indicate the protein spots with altered abundance.

To identify novel substrates of PI3Kγ that play a role in MDA-MB-231 cell migration upon IGF-I-induced IGF-1R-CXCR4 transactivation, a 2D DIGE proteomic approach was employed. To identify proteins that are regulated by PI3Kγ, with a particular focus on phosphorylation, control and PI3Kγ knockdown cells were treated with or without IGF-I for 5 minutes, a time point at which PI3Kγ is maximally active in this system (as shown by phosphorylation of Akt as a readout for PI3Kγ activity), and the cytosolic fraction was collected. For each tested condition, triplicate biological replicates were obtained and reverse-labeled with Cy3 or Cy5 while the Cy2 dye was used for the internal standard control for normalisation and quantitation of the Cy3- and Cy5-labeled samples. The samples were combined and resolved on 2D gel electrophoresis and proteins were analysed using DeCyder 2D software. According to the Decyder software analysis, about 427 protein spots were visualized, 10 of which exhibited differences in protein abundance between the control and p110γ knockdown cells under resting conditions (Figure 3A+B). The proteomic analysis after IGF-I stimulation showed that about 1207 protein spots in the one 2D-gel were detected, 38 of which exhibited alterations in protein abundance in the absence of p110γ (Figure 3C+D). Protein abundance changes were considered significant using a two-tailed Student’s t-test p-value of less than 0.05. For identified abundance changes, fold-changes between the control and p110γ knockdown cells without and with IGF stimulation are listed in Tables 1 and 2.

**Table 1:** **List of differentially-expressed proteins between control and p110γ knockdown cells without IGF-I stimulation determined by DIGE and identified by MS**^**a**^

**Name**	**Accession**	**MW (kDA)/pI**	**Spot No.**	**Fold change**	**Mascot search results**		
					**ID/Total queries**	**Sequence coverage (%)**	**Combined IonScore**
Pyruvate kinase isozyme M1/M2	KPYM_HUMAN	58.5/7.96	122	1.2	62/566	49	1121
Phosphoglycerate kinase 1	PGK1_HUMAN	45.0/8.30	222	1.5	39/540	44	607

^a^All other differentially-regulated spots were identified as BSA or keratin.

**Table 2:** **List of differentially-expressed proteins between control and p110γ knockdown cells after 5 Min IGF-I stimulation determined by DIGE and identified by MS**^**a**^

**Name**	**Accession**	**MW (kDA)/pI**	**Spot No.**	**Fold change**	**Mascot search results**		
					**ID/Total queries**	**Sequence coverage (%)**	**Combined IonScore**
Eukaryoticelongation factor 2	eEF2_HUMAN	96.2/6.4	100	1.4	22/545	18	395
			101	1.3	14/547	16	215
			106	1.3	10/534	9	137
Pyruvate kinase isozyme M1/M2	KPYM_HUMAN	58.5/8.0	265	1.3	45/462	41	882
			267	1.3	52/560	47	1112
			275	1.3	78/572	69	1471
Alpha-enolase	ENOA_HUMAN	47.5/7.0	449	1.2	27/481	45	556
Phosphoglycerate kinase 1	PGK1_HUMAN	45.0/8.3	542	1.2	17/519	34	387
L-lactate dehydrogenase A chain	LDHA_HUMAN	37.0/8.4	734	1.4	11/603	23	185
			746	1.4	8/507	15	112
Purine nucleoside phosphorylase	PNPH_HUMAN	32.3/6.4	837	−1.3	3/585	9	63

^a^ All other differentially-regulated spots were identified as BSA or keratin.

**Figure 4:**
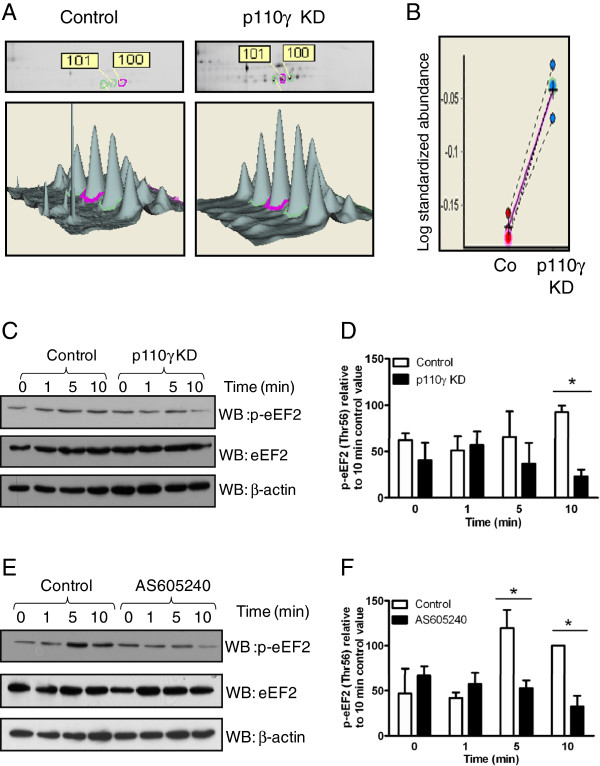
**Phosphorylation of eEF2 in response to IGF-I is PI3Kγ-dependent in MDA-MB-231 cells.** (**A**) Spot map and three-dimensional view of differentially expressed proteins, the spot circled with pink line represents spot 101. (**B**). Graph view of spot 101. (**C**) Control or p110γ knockdown MDA-MB-231 cells were incubated in serum-free media for 1 hour before being stimulated with 0.1 nM IGF-I. Cell lysates were prepared and subjected to SDS-PAGE and Western blot to detect phosphorylated eEF2. Membranes were stripped and reprobed for eEF2 and β-actin as loading controls. These data are representative of at least 3 independent experiments conducted with similar results. (**D**) eEF2 phosphorylation was quantified by densitometry and normalized to the level of β-actin and expressed as a value relative to the 10 minute control-treated values (mean ± SEM of three independent experiments). * - significantly different from control values (2-way ANOVA with Bonferroni post-test) at *, p<0.05. (**E**) MDA-MB-231 cells were either untreated or treated with 2μM AS605240 and incubated in serum-free media for 1 hour and stimulated with 0.1 nM IGF-I. Cell lysates were prepared and subjected to SDS-PAGE and Western blot to detect phosphorylated eEF2. These data are representative of at least 3 independent experiments conducted with similar results. (**F**) eEF2 phosphorylation was quantified by densitometry, normalized to β-actin and expressed as a value relative to the 10 minute control-treated values (mean ± SEM of three independent experiments). * - significantly different from the control values (2-way ANOVA with Bonferroni post-test) at *, p<0.05.

LC-ESI-MS/MS was applied to identify the differentially-expressed proteins between the control and p110γ knockdown cells. The Mascot search results are detailed in Tables 1 and 2 for each identification. Two of the proteins identified by MS were regulated by p110γ under both IGF-I and non-IGF-I stimulation conditions: Pyruvate kinase isozyme M1/M2 (KPYM) and Phosphoglycerate Kinase 1 (PGK1). Four of the proteins identified were regulated by p110γ exclusively after IGF-I stimulation: Alpha-enolase (ENOA), L-lactate dehydrogenase A chain (LDHA), Purine nucleoside phosphorylase (PNPH) and eukaryotic elongation factor 2 (eEF2). All other differentially-regulated spots were identified as BSA or keratin,which were most likely added through unavoidable contamination. Three of the identified proteins are involved in metabolism, while eEF2 is known as a regulator of protein synthesis. Because of the previous implication of other PI3Ks in eEF2 activity [21], we focused our attention on this protein. Enlarged regions of images and three-dimensional fluorescence intensity profiles of a representative spot 101 identified as eEF2 is shown in Figure 4A. All biological replicates showed a similar increase in abundance (Figure 4B). The characteristics of the abundance of this protein as well as the short stimulation time used in the experiment strongly suggest that the difference observed is due to a posttranslational modification, and based on the shift from the acidic to basic site of the gel, it is likely that the protein is less phosphorylated in the p110γ knockdown cell line when compared to the control cells.

### Phosphorylation of eukaryotic Elongation factor 2 (eEF2) in response to IGF-I is PI3Kγ-dependent

To confirm the involvement of the regulation of eEF2 downstream of PI3Kγ, control and PI3Kγ knock-down cells were stimulated with IGF-I and the lysates were immunoblotted with anti-phospho-eEF2, followed by stripping and reprobing of the western-blot with anti- eEF2 antibody. The results demonstrated that the phosphorylation of eEF2 was decreased in p110γ knockdown cells compared to that in the control cells, whereas the total eEF2 protein was not affected (Figure 4C and densitometry analysis 4D). Although the phosphorylation of eEF2 was initially shown to be significantly different after 5 min of stimulation, the response seems to be more reproducible and robust after 10 min of stimulation.

In addition, the lysates from parental MDA-MB-231 cells pre-treated with the isoform specific inhibitor, AS605240, followed by stimulation with IGF-I were immunoblotted with phospho-eEF2 and total eEF2 antibodies. The results of these experiments showed that the level of phospho-eEF2 in response to IGF-I was significantly decreased after AS605240 treatment compared with control cells (Figure 4E and densitometry analysis 4F). Taken together, these data indicate that eEF2 is phosphorylated downstream of activation of the IGF-1R-CXCR4 heterodimer in response to IGF-I and that this is dependent on PI3Kγ activation.

## Discussion

Activation of receptor tyrosine kinases (RTKs) and G-protein coupled receptors GPCRs by their ligands leads to the activation of intracellular signaling cascades. While these pathways were initially thought to be distinct, recent data indicate an important role for RTK-GPCR transactivation in a number of physiological and pathological cellular responses. This form of receptor transactivation has been shown to regulate cell proliferation [22-24], migration and invasion [1,25,26] in various types of cancer, and our recent data indicate an important role for IGF-1R and CXCR4 transactivation in migration of MDA-MB-231 breast cancer cells [2]. This IGF-1R-CXCR4 heterodimer appears to be linked with the metastatic phenotype of these cells as the related but non-metastatic MCF-7 breast cancer cell line does not express functional heterodimers. Therefore, it is important to understand how IGF-1R-CXCR4 transactivation facilitates migration of MDA-MB-231 cells. In the present study, three novel observations with respect to IGF1-R and CXCR4 transactivation were made. First, PI3Kγ is the major PI3K isoform involved in IGF-I-induced cell migration of the metastatic breast cancer cell line MDA-MB-231. Second, eEF2 is one of the downstream targets of PI3Kγ after this heterodimeric receptor transactivation. Third, IGF-1R-CXCR4 transactivation leads to PI3Kγ-dependent phosphorylation of eEF2. These findings indicate that PI3Kγ may promote breast cancer cell migration through a novel mechanism by deactivating eEF2 after IGF-1R-CXCR4 transactivation.

Activation of the class IA PI3Ks, PI3Kα, β and δ following ligation of IGF-1R by IGF-1 is well documented [27-30]**.** However, the two major PI3K isoforms known to be activated downstream of GPCRs and to play a role in cell migration in response to GPCR ligands are p110γ and p110δ [4,9,31-33]. Thus, we investigated the expression of these PI3K isoforms in metastatic MDA-MB-231 and observed that these cells express both p110γ and p110δ. Our previous data indicate that MDA-MB-231 cells express a functional IGF-R-CXCR4 heterodimer whereas MCF-7 cells do not [2]. In fact, IGF-I signals directly through IGF-1R in MCF-7 cells to control migration of the cells, independently of CXCR4 [2]. We therefore investigated the level of expression of these PI3K subunits in MCF-7 cells and found that while both MCF-7 and MDA-MB-231 cells express similar levels of p110δ, MCF-7 cells express a low level of p110γ.

PI3Kγ is generally activated by GPCRs, including chemokine receptors, such as CXCR4 [4], but to the best of our knowledge, has not been implicated in IGF-1R signaling. Here, we show that IGF-I stimulation leads to the membrane translocation of p110γ, an indicator of PI3K activation. Moreover, specific inhibition of PI3Kγ and knockdown of p110γ resulted in decreased phosphorylation of Akt and cell migration in response to IGF-I, whereas PI3Kδ did not appear to be involved in this response. Taken together, these data indicate that PI3Kγ is a major PI3K isoform regulating MDA-MB-231 breast cancer cell migration in response to IGF-I.

To shed light on the signaling pathways regulated by p110γ downstream of IGF-1R-CXCR4 transactivation, we performed a 2D DIGE proteomics experiment. We compared the cytosolic proteome from MBA-MB-231 control cells with MBA-MB-231 cells in which p110γ has been knocked down after 5 min of IGF-I stimulation. Importantly, this short stimulation time allowed us to focus on post-translational modifications to the MDA-MB-231 cell proteome as this time point was too short for effects on gene expression. These experiments identified eEF2 as one of the downstream effectors of PI3Kγ after receptor transactivation. eEF2 is known to play a critical role in regulating protein synthesis by mediating the ribosomal translocation from the A to the P-site in eukaryotic tissues, the reaction that induces movement of mRNA along the ribosome during translation [34]. Phosphorylation of eEF2 prevents functional binding to the ribosome and delays the elongation step, thereby terminating translation [35]. PI3Ks have previously been implicated in the regulation of the eEF2 downstream of proliferative signals [21], however whether specific PI3K isoforms or all PI3K isoforms regulate eEF2 signaling has not yet been determined.

Regulation of the eEF2 activity by PI3Kγ may be the result of multiple molecular mechanisms. Firstly, PI3Kγ may activate the eEF2 kinase through the mTOR/P70S6K pathway, which has been shown as a key pathway regulating eEF2 activity [21]. Secondly, PI3Kγ may reduce the rate of eEF2 dephosphorylation through inhibiting the activity of a protein phosphatase such as protein phosphatase 2A (PP2A) [36,37]. The first protein kinase substrate of PI3Kγhas been identified recently. PI3Kγphosphorylates SET, an endogenous inhibitor of PP2A, on two serine residues. eEF2 might therefore be a direct or indirect substrate of PI3Kγ.

Cells can respond to growth factors by either migrating or proliferating, but not both at the same time, a phenomenon termed “migration-proliferation dichotomy” [38], This is not only observed in cancer progression but also during wound healing and developmentand the underlying mechanism remains unknown. The proposed physiological basis for this phenomenon is that directional cell migration occurs along an increasing ligand gradient until migrating cells reach a zone in which they start dividing as a result of the presence of ligands that regulate proliferation. Thus limited protein synthesis occurs in migrating cells, which diverts energy to the process of migration. However, when cells stop migrating and start proliferating, protein synthesis is necessarily upregulated. Our results support the view that reduced proliferation is an integral part of migration and, more specifically, that in metastatic breast cancer cells the initiation of both processes might be regulated by PI3Kγ.

It should be noted that the data presented in this manuscript have been obtained using one breast cancer cell line and further experimentation will be required to determine the generality of our observation. This would include examining different cell lines (from breast and other cancers) and ideally, in cells from clinical tissue samples. With respect to the latter, unfortunately at present there is no means by which to identify cells in tissues in which IGF1R/CXCR4 transactivation occurs. However, ultimately, an improved understanding of the molecular mechanisms underlying IGF1R/CXCR4 transactivation, including the role of PI3K signal transduction pathways, in the progression of breast cancer metastasis and invasion may lead to development of more effective diagnostic and therapeutic strategies.

We have also observed that phosphorylation of eEF2 occurs upon stimulation of MDA-MB-231 cells with CXCL12 (the chemokine ligand of CXCR4) in a PI3Kγ-dependent manner (data not shown). Future studies could determine if phosphorylation of eEF2 generally occurs downstream of activated G-protein coupled receptors. Importantly, the results of a recent study implicate phosphorylation of eEF2 as an important link between the DNA damage response and translation of mRNAs [16]. After activation of the DNA damage checkpoint, AMPK mediates activation of eEF2 kinase, which in turn phosphorylates eEF2. The authors conclude that because protein synthesis is energetically costly, stressed cells inhibit this process to devote resources to the stress response. That study, together with the observations in the present study, implies that phosphorylation of eEF2 to inhibit translation may be a general mechanism regulating energy consumption between important energy-dependent cellular processes.

## Conclusion

In summary, we provide novel mechanistic data further characterizing the downstream signalling pathways elicited upon activation of the IGF-1R-CXCR4 heterodimer in metastatic MDA-MB-231 breast cancer cells. Our findings indicate that PI3Kγ may promote breast cancer metastasis through a novel mechanism, by deactivating eEF2 after IGF-1R-CXCR4 transactivation.

## Methods

### Cell lines and treatment conditions

Human breast cancer cell lines, the non-metastatic MCF-7 and highly metastatic MDA-MB-231 cells, were obtained from American Type Culture Collection (ATCC; Manassas, VA, USA) [2,3]. The MDA-MB-231 p110γ knockdown cells were generated by lentiviral transductions using shRNA constructs in pLKO.1 (Open Biosystems Inc, Huntsville, USA) according to the manufacturer’s instructions. The knockdown of p110γ was confirmed by Western-blot analysis [17]. MCF-7 cells were cultured in Dulbecco’s modified Eagle’s medium (Gibco, USA) supplemented with 10% fetal bovine serum whereas MDA-MB-231 cells were cultured in RPMI-1640 (Gibco, USA) with 10% fetal bovine serum. For IGF-I or inhibitor treatment, cells were incubated in serum-free medium supplemented with 0.5% BSA (Sigma-Aldrich, St Louis, MO, USA) for 1 hour.

### Reagents

IGF-I was obtained from GroPep Pty Ltd (Adelaide, SA, Australia). AS605240 was from Echelon Biosciences Inc (Salt Lake City, UT, USA). IC87114 was from Australian Centre for Blood Diseases (Monash University, Australia). Anti-human Phosphoinositide 3-kinase γ, anti-phosphorylated-Akt (S473), anti-Phospho-eEF2 (Thr56) and anti-eEF2 antibodies were purchased from Cell Signaling Technology (Danvers, MA, USA). Anti-pan-cadherin and anti-β-actin antibodies were obtained from Sigma-Aldrich (St Louis, MO, USA). Rabbit anti-p110δ antibodies were produced from peptides using standard immunization. The immunizing peptide was KVNWLAHNVSKDNRQ.

### Membrane fractionation

The cells were washed, scraped, and suspended in hypotonic buffer (10 mM Tris–HCl (pH 7.9) containing 1.5 mM MgCl_2_, 10 mM KCl, 0.5 mM dithiothreitol, 0.1% Nonidet P-40, and protease inhibitors); incubated on ice for 10 min; homogenized with 20 strokes of a glass Dounce homogenizer; and centrifuged at 500 × *g* for 5 min at 4°C to yield the nuclear fraction. The nuclear fraction was then suspended in 200 μl of extraction buffer (20 mM Tris–HCl (pH 7.9) containing 20% glycerol, 1.5 mM MgCl_2_, 0.5 mM dithiothreitol, and protease inhibitors), and 4 M KCl was added to a final concentration of 0.3 M. The final suspension was rocked for 30 min at 4°C and centrifuged at 13,000 × *g* for 15 min to yield the nuclear fraction. The 500 × *g* post-nuclear supernatant fraction was further fractionated by centrifugation at 100,000 × *g* for 1 h at 4°C. The resulting pellet was dissolved in 5-fold Laemmli buffer and designated as the membrane fraction.

### Immunoprecipitation and western blot analysis

Cells were lysed in lysis buffer (50 mM Tris [pH 7.5], 1% [wt/vol] NP-40, 150 mM NaCl, 1 mM ethylene diamine tetraacetic acid (EDTA), 1.5 mM MgCl_2_, 50 mM NaF, 1 mM Na_3_VO_4_, 1 mM phenylmethylsulfonyl fluoride) and 1% protease inhibitors (Sigma, USA) on ice for 30 min. The lysates were centrifuged at 13,000 × g for 10 min at 4°C. The supernatant was collected and the protein concentration was determined using the BCA protein assay (Pierce). For immunoprecipitation, the lysates (1 mg of total protein) were incubated with 1 μg of anti-p110γ, at 4°C overnight. Immunocomplexes were precipitated with protein A-sepharose beads at 4°C for 1 h. After three washes with lysis buffer, the bound proteins were eluted from the column in preheated sample buffer (50 mM Tris–HCl pH 6.8, 50 mM dithiothreitol, 1% SDS, 0.005% bromphenol blue, and 10% glycerol). For whole lysate sample preparation, the lysates (50 μg of total proteins/well) were denatured by boiling for 5 min in sample buffer. The immunoprecipitates and whole lysates were then subjected to 10% SDS-PAGE, transferred to PVDF membrane (Millipore, USA), and analyzed by Western blotting.The transferred membranes were blocked with 5% skim milk powder and incubated with primary Abs (1:1000 of anti-phosphorylated-Akt (S473), 1:1000 of anti-Phospho-eEF2, 1:1000 of anti-eEF2, 1:1000 of anti-pan cadherin, 1:500 of anti-p110γ, 1:5000 of anti-β-actin) overnight at 4°C followed by horseradish peroxidase-conjugated goat anti-rabbit IgG (1:50000) or horseradish peroxidase-conjugated goat anti-mouse IgG (1:1000). Membranes were visualized by enhanced chemiluminescence (Sigma, USA). Membranes were stripped with Restore™ Western Blot Stripping Buffer (Pierce, Rockford) according to the manufacturer’s instructions.

### Chemotaxis assay

Chemotaxis was measured in a modified Boyden Chamber as described previously [2].

### Preparation of protein samples and 2D-DIGE

Control and p110γ knockdown MDA-MB-231 cells either unstimulated or stimulated with IGF-I for 5 minutes were lysed in hypotonic lysis buffer (10 mM Hepes, pH 7.9, 133 mM sorbitol, containing 5 mM NaF, 2 mM Na_3_VO_4_, 1 mM PMSF and protease inhibitor (1:100, Sigma-Aldrich) for 10 min at 4°C, homogenized, and then spun at 800 × g for 10 min. The pellet was washed with the hypotonic buffer and the supernatants were combined to generate the cytosolic fraction. These samples were then precipitated with a Clean-up kit (GE Healthcare, UK) and suspended in labeling buffer (7 M Urea, 2 M Thiourea, 4% (w/v) CHAPS, 30 mM Tris, pH 8.5). Protein concentrations in the control and PI3Kγ knockdown cell lines were determined by an EZQ protein quantitation assay (Invitrogen/Molecular Probes) against an ovalbumin standard curve according to the manufacturer’s instructions. Each of the tested conditions (resting and IGF-I-stimulation) was repeated in triplicate. Protein from each sample was labeled according to the manufacturer’s instructions (GE Health care) with CyDyes (Cy2, Cy3 and Cy5). Briefly, 50 μg of protein from each sample were labeled with 200 pmol of either Cy3 or Cy5 and a reverse-labeling approach was used to avoid dye-labeling bias. The gel-to-gel variation was excluded by using an internal protein standard (IPS)sample, obtained by mixing equal amounts of proteins from the test conditions. As common practice, the Cy2 minimal dye (GE Healthcare) was used to label the IPS prepared by pooling 25 μg of protein from each sample (150 μg of total protein).

Six individual samples from the control and p110γ knockdown cells under each of the test conditions (control and IGF-I-stimulation) were co-resolved in 6 different 2D-DIGE gels. Isoelectric focusing (IEF) was performed on an immobilized non-linear pH 3–11 gradient of 24 cm length (GE Healthcare), using an Ettan IPGphor II system (GE Healthcare) with the current limited to 50 μA per strip. Following IEF, the strips were equilibrated in equilibration buffer (Gel Company) with 6 M urea added, containing 10 mg/ml of DTT for 15 minutes followed by the exchange of solution for equilibration buffer that contained 25 mg/ml of iodoacetamide (IAA) in place of DTT. SDS-PAGE in the second dimension was carried out using 12.5% 2D gel DALT NF precast polyacrylamide gels (Gel Company). Electrophoretic separation was performed using an Ettan Dalt 12 Separation Unit (GE Healthcare) in the electrophoresis buffer provided with the pre-cast gels at 25°C using the following conditions: 50 V, 5 mA/gel, 0.5 W/gel, for 1 hour, 110 V, 10 mA/gel, 0.5 W/gel, for 1 hour, 250 V, 30 mA/gel, 2.5 W/gel until the dye-front emerged from the bottom of the gel. Gel images of Cy2, Cy3, and Cy5 were scanned by using Typhoon Trio at 100 μm resolution (GE Healthcare). To assure that the image was not saturated, the PMT voltage (550 V) was altered, that the pixel intensities in the scanned image was below 80,000 counts.

### Spot detection, quantification and comparisions

Image analysis was undertaken using DeCyder 2D software (version 7, GE Healthcare). The control and p110γ knockdown samples were compared using a two-tailed Students t-test to detect spots that were differentially expressed. Those spots that returned a p-value of <0.05 were accepted and selected for protein in-gel digest, LC-eSI-I MS/MS analysis, and identification.

### LC-ESI-MS/MS

Protein spots showing statistically significant differences in expression between control and p110γ knockdown cells were excised from the gel using an Ettan Spot Picker (GE Healthcare), reduced, alkylated and digested using trypsin (100 ng of sequencing grade modified trypsin (Promega) in 5 mM ammonium bicarbonate in 10% Acetonitrile (ACN)). After extraction with 1% formic acid (FA) in water, 1% FA in 50% ACN and 100% ACN, the volumes of the resulting peptide extracts were reduced by vacuum centrifugation to approximately 1 μL. Vacuum concentrated samples were resuspended with 0.1% FA in 2% ACN to total volume of ~8 μL. LC-ESI-IT MS/MS was performed using an online 1100 series HPLC sytem (Agilent Technologies) and HCT Ultra 3D-Ion-Trap mass spectrometer (Bruker Daltonics). The LC system was interfaced to the MS using an Agilent Technologies Chip Cube operating with a ProID-Chip-150 (II), which integrates the enrichment column (Zorbax 300SB-C18, 4mm, 40 nL), analytical colum (Zorbax 300 SB-C18, 150 mm × 75 μm), and nanospray emitter. Five microlitres of sample was loaded on the enrichment column at the flow rate of 4 μL/min in mobile phase A (0.1% FA in 98% m/v ACN) over 32 min at 300nL/min. Ionizable species (300< m/z <1,200) were trapped and the most intense ions eluting at the time were fragmented by collision-induced dissociation. Active exclusion was used to exclude a precursor ion for 30 seconds following the acquisition of two spectra.

MS and MS/MS spectra were subjected to peak detection and de-convolution using DataAnalysis (Version 3.4, Bruker Daltonics, Billerica, MA, USA). Compound lists were exported to mascot generic format (mgf) and submitted to theMASCOT database-searching engine (Version 2.2, Matrix Science, Boston, MA, USA). The search parameters were as follows: Swissprot release 57.7, Taxonomy Mammalia (64799 entries), Enzyme: Trypsin (2 missed cleavages), Fixed modifications: Carbamidomethyl (C), Variable modifications: Oxidation (M), N-terminal Glutamine to pyro-glutamic acid, N-terminal Glutamic acid to pyro-Glutamic acid; Mass tolerances MS 0.3 Da, MS/MS: 0.4 Da. Protein identifications were made on the basis of having at least two matching unique peptides with individual ion scores above the mascot 0.05 significance threshold (95% confidence).

### Quantitation of western blot densitometry

Gelbands were quantified usingImagequantsoftware (GE Healthcare, USA). All values were normalized to the appropriate loading control (β–actin) and then expressed as a value relative to the 10 minute control-treated values essentially as previously described [39].

### Statistical analysis

Statistical analyses were conducted by unpaired Student’s t-tests analysis or 2-way ANOVA with Bonferroni post-tests, as stated in the figure legends, using GraphPad Prism software (GraphPad Software, Inc, USA).

### Design of the study

The experimental design is outlined in Additional file 1: Figure S1.

## Abbreviations

DIGE: Differential gel electrophoresis; GPCR: G-protein coupled receptor; IEF: Iso electric focusing; IGF: Insulin-like growth factor; PI3K: Phosphatidyl-inositol-3 kinase; RTK: Receptor tyrosine kinase.

## Competing interests

The authors declare that they have no competing interests.

## Authors’ contributions

MN performed most of the experiments, interpreted the data and drafted the manuscript. JAB, BF, CA and PH have made significant contribution to experimental design, data analysis and/or interpretation of the data. MKH and SRM conceived and designed the study, designed the experiments, interpreted the data and wrote the manuscript. All the authors have read and approved the final manuscript.

## Supplementary Material

Additional file 1: Figure S1 Work flow used in this study. Click here for file
