# Supplementary material for: Comparative proteomic analysis implicates eEF2 as a novel target of PI3Kγ in the MDA-MB-231 metastatic breast cancer cell line
Source: Proteome Sci. 2013 Jan 15;11:4. doi: 10.1186/1477-5956-11-4 (PMC3564858; doi:10.1186/1477-5956-11-4)
Supplement: Additional file 1: Figure S1 — Work flow used in this study. [file 1477-5956-11-4-S1.ppt]

## Slide 1
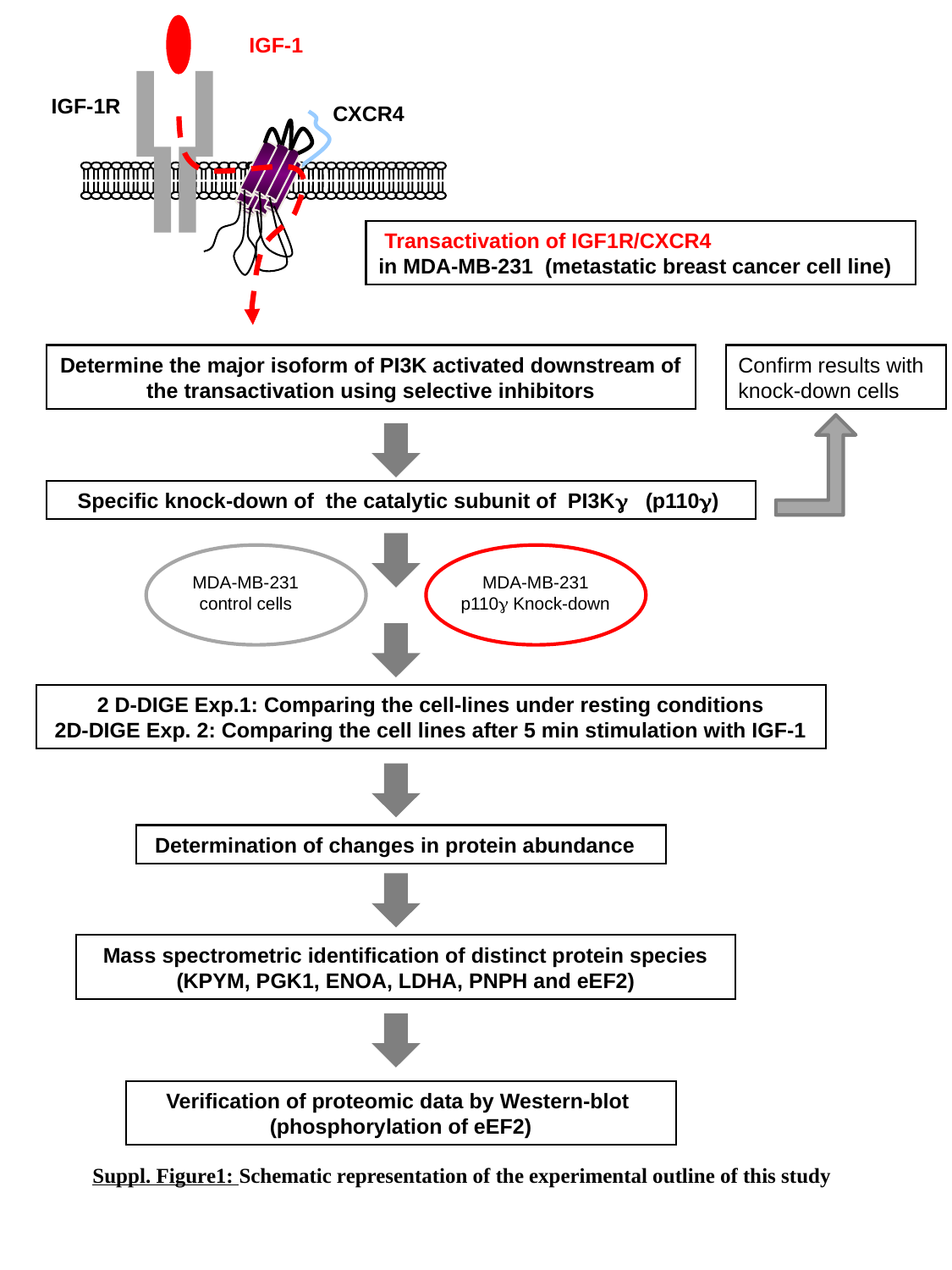

IGF-1
IGF-1R
CXCR4
 Transactivation of IGF1R/CXCR4
in MDA-MB-231 (metastatic breast cancer cell line)
Determine the major isoform of PI3K activated downstream of the transactivation using selective inhibitors
Confirm results with knock-down cells
Specific knock-down of the catalytic subunit of PI3Kγ (p110γ)
MDA-MB-231
control cells
MDA-MB-231
p110γ Knock-down
2 D-DIGE Exp.1: Comparing the cell-lines under resting conditions
2D-DIGE Exp. 2: Comparing the cell lines after 5 min stimulation with IGF-1
Determination of changes in protein abundance
Mass spectrometric identification of distinct protein species (KPYM, PGK1, ENOA, LDHA, PNPH and eEF2)
Verification of proteomic data by Western-blot
(phosphorylation of eEF2)
Suppl. Figure1: Schematic representation of the experimental outline of this study
